# The Application of External Ureteral Catheters in Children With Acute Kidney Injury Caused by Ceftriaxone-Induced Urolithiasis

**DOI:** 10.3389/fped.2020.00200

**Published:** 2020-04-22

**Authors:** Houwei Lin, Hongquan Geng, Guofeng Xu, Xiaoliang Fang, Lei He, Maosheng Xu

**Affiliations:** ^1^Department of Pediatric Urology, Xinhua Hospital, Shanghai Jiao Tong University of Medicine, Shanghai, China; ^2^Children's Urolithiasis Treatment Center of Chinese Health Committee, Shanghai, China

**Keywords:** external ureteral catheter, acute kidney injury, ceftriaxione, urolithiasis, children

## Abstract

**Objective:** To evaluate our use of external ureteral catheters in children with acute kidney injury (AKI) resulting from ceftriaxone-induced urolithiasis.

**Methods:** From July 2010 to June 2015, a series of 15 children, including 12 males and 3 females, were referred to our department. All of them were diagnosed of post-renal AKI and underwent emergent hospitalization. Evaluation of serum electrolytes, creatinine (Cr), blood urea nitrogen (BUN), complete blood count, and blood gas analysis were completed in each child both before they were admitted, and again after surgery. Bilateral externalized ureteral catheters were placed cystoscopically in each of these patients. The composition of collected calculi was analyzed by infrared spectrography.

**Results:** Bilateral externalized ureteral catheters were placed successfully in all patients. There were no procedure-related complications. Two days after catheter placement, the levels of serum Cr and BUN had improved in all patients, and these levels were noted to be significantly lower than before catheterization (*P* < 0.001). Infrared spectrography demonstrated that the primary composition of all calculi collected was ceftriaxone. No recurrent AKI or renal deterioration was detected during the follow-up which ranged from 3 to 8 years.

**Conclusions:** These results show that short-term external ureteral catheters can be effectively utilized in children with AKI caused by ceftriaxone-induced urolithiasis. We recommend this procedure as a viable replacement to indwelling stents in patients with bilateral ureteral stones.

## Introduction

One of the most common causes of urinary tract obstruction and postrenal acute kidney injury (AKI) is ureteral calculi. In a previous manuscript we reported a series of children who had developed AKI after developing bilateral ureteral obstruction from ceftriaxone calculi (1). Emergent intervention is, of course, required for such children. Ureteroscopy (URS) is the most common endoscopic method for treating ureteral stones but can be difficult, and sometimes impossible, at the time of the first intervention. It is therefore often necessary in children, to first place double J ureteral stents for a period of time, allowing for passive ureteral dilation, prior to proceeding to URS and stone removal. Many pediatric urologists will then leave double J ureteral stents in place again after URS, for a period of time, to allow procedural trauma and edema to heal. Since children often cannot tolerate office-based cystoscopy and stent removal, final stent removal may be performed in the operating room with general anesthesia. So, some children will require a total of three separate general anesthesia-based procedures to treat the ureteral stone. While the use of double J ureteral stents has been shown to be safe and effective by various studies (2–5), this treatment protocol can be rather lengthy and difficult for patients. In addition, it can be difficult to place double-J stents for some children. Additionally, there are a variety of complications related to the use of double-J ureteral stents. In this article, we describe our experience using external ureteral catheters instead of double-J stents, as a viable option for relieving bilateral ureteral obstruction in children who suffer from AKI caused by ceftriaxone-associated urolithiasis.

## Methods

### Patients and Symptoms

We performed a retrospective review of 15 children that suffered from AKI. From July 2010 to March 2015, 15 patients (12 male and 3 female) were referred to our department, the Pediatric Urology Department at Xin Hua Hospital, affiliated with Shanghai Jiaotong University School of Medicine. The patients' mean age was 3.76 ± 2.74 years (range 5 months to 11 years). Within several days of presentation, all children had received ceftriaxone therapy at a dose of 1 g once daily, which is equivalent to the adult dose. No other risk factors of stone formation could be identified. Neither urinary tract abnormality nor a family history of urolithiasis was found in any of the patients.

The main clinical manifestation of anuria was present in nine patients for ~10 h to 3 days. Six patients had a history of oliguria for ~1–7 days; oliguria is defined as a urine output <1 mL/kg/h in infants or <0.5 mL/kg/h in children. We estimated the urine output volume of the children based on the description of the parents.

### Examinations

An abrupt reduction in kidney function is currently defined as a ≥50% increase in serum creatinine or the occurrence of oliguria (5). But under our emergency situation, we didn‘t have time to repeat the examination before surgery. So our diagnosis of AKI was based on sudden onset of anuria or oliguria associated with elevated serum creatinine in a child who had no past history of urolithiasis. Serum electrolytes, serum creatinine, blood urea nitrogen (BUN), a complete blood counts, and blood gases were evaluated in every child before and after surgery. Urinalysis and urine culture were performed after the operation; preoperative urinalysis was not performed because of insufficient urine volume. All patients underwent ultrasound before and after surgery. Kidney, ureter and bladder (KUB) X-rays and CT were performed in some patients. All stones, which were collected intraoperatively, were analyzed by infrared spectrometer (Tensor 27, Bruker AXS company, Germany). Single photon emission computed tomography (SPECT) was used to evaluate the effective renal plasma flow (ERPF) and split renal function of bilateral kidneys during follow-up.

### External Ureteral Catheterization Procedure

After the diagnosis was established, emergency intervention was carried out without delay. General anesthesia was employed in all patients during the procedure. Under cystoscopy, we inserted a 4-Fr or 5-Fr ureteral catheter at the opening of one ureter and gradually entered it until we felt resistance. We gently increased the pushing force and let the catheter move forward. Then we would feel the resistance suddenly disappear and a cloud of bloody mixture poured from the opening of the ureter (Video S1). At that time, we checked the end of the catheter, if you see a continuous urine outflow (Video S2), it meant that the tube had passed through the obstruction zone, demonstrating relief of the obstruction. The same way we inserted another catheter on the other side. Then we fixated the external stents to an urethral catheter so that it would not dislocate. As a result, the children had limited mobility. We attached a bag to each catheter in order to observe the drainage of each catheter. The urinary sediment was collected from the urine bags, and the stones were analyzed. None of the patients underwent continuous renal replacement therapy (CRRT), PCNL, ureteroscopy, or double–J stent insertion.

### Removal of External Ureteral Catheter

Successful calculi eradication was defined as complete clearance of stones as evidenced by ultrasonography. Patients were also considered stone-free if residual stones after treatment were smaller than 2 mm, defined as clinically insignificant residual fragments. We removed the external ureteral catheters when the ultrasound confirmed the stone-free, usually 2–3 days after catheterization.

### Statistical Analysis

Statistical analysis was performed using SPSS, version 19.0. Data are presented as mean ± standard deviation; differences were analyzed using the Student's *t*-test. A *p* < 0.05 was considered statistically significant.

## Results

Multiple calculi in the upper urinary tract was identified with all patients. Two patients were diagnosed with bilateral renal calculi. Three of them had bilateral ureteral stones and bilateral renal calculi. Nine of them had bilateral ureteral stones, and one of them had unilateral ureteral calculi and contralateral renal calculi. Mild to moderate hydronephrosis was found bilaterally in all patients.

Acoustic shadows at the rear and trailing edge were detectable by ultrasonography. KUB X-ray of all examined patients showed no stone-like shadows. The CT values of stones of all examined patients were relatively low (mean, 144.09 ± 50.09 Hounsfield Units).

All of the patients had successful external ureteral catheterization procedures. Two days after treatment, the levels of serum creatinine and BUN showed clear improvement and were significantly lower than preoperative levels (*p* < 0.001). In addition, serum potassium (K+) had also significantly improved relative to pre-treatment values (*p* < 0.05); these parameters are shown in Table 1. The urine cultures of all patients were negative.

**Table 1:** The results of blood biochemical indexes before and after treatment.

**Time**	**K (mmol/L)**	**Na (mmol/L)**	**BUN (mmol/L)**	**Cr (umol/L)**
Before treatment	5.22 ± 1.10	134.92 ± 3.50	22.72 ± 10.77	355.46 ± 193.05
Two days after treatment	3.98 ± 0.28	135.08 ± 3.55	4.61 ± 2.32	38.00 ± 18.02
*P*-value	<0.001	0.91	<0.001	<0.001

Most of the calculi was flushed away during the catheterization, so we could only collect four samples of urinary calculi. The results of infrared spectrometry showed that the main calculus composition was ceftriaxone. No severe intraoperative complications, such as ureteral perforation, catheter breakage, or mucosal avulsion, occurred. All of the external ureteral catheters were removed when ultrasound examination could confirm stone-free after treatment.

No renal deterioration was detected among our group of discharge. All patients were followed up for 3–8 years. No recurrent AKI was found, and ultrasonography showed that none of the patients had urolithiasis or hydronephrosis. Renal isotope scans (SPECT) were performed to assess patients' excretory function and split renal function of their follow-ups. All patients showed good excretory function and normal split renal function 6 months after hospital discharge.

## Discussion

A recent assessment of the incidence of urolithiasis in children reported 50 cases per 100,000 children (6). Moreover, the incidence of pediatric urolithiasis is increasing in China. The majority of these stones are composed of calcium—predominantly calcium oxalate but also, to a lesser extent, calcium phosphate. Much less commonly, calculi is composed of urate, cysteine, or struvite (7). Although it is rare that the stones are composed of ceftriaxone calcium, ceftriaxone has been associated with renal, and ureteral calculi in adult and pediatric populations, as demonstrated by our previous report and other studies (1, 8–10).

Our recent study also revealed that this type of stone tends to obstruct the ureters bilaterally, which rapidly leads to AKI. Undoubtedly, AKI is an emergency condition that can threaten the lives of patients. Thus, we prefer relieving the obstruction or urinary diversion surgically, using techniques such as placing indwelling double-J stents by cystoscopy, ureteroscopy, percutaneous nephrolithotomy, or open surgery, rather than administering dialysis (e.g., CRRT). The placement of indwelling double-J stents is most often chosen to relieve bilateral ureteral obstruction because it is a fast and convenient procedure that induces minimal damage. However, ureteroscopy, percutaneous nephrolithotomy, or even open surgery are necessary if the insertion of double-J stents via cystoscopy fails. Additionally, indwelling double-J stents remained *in situ* for several weeks postoperatively, which necessitates a second operation and can cause duration-dependent complications like stent colic and hematuria. In our population parents are relucatnat to subject children to successive anesthetic sessions, therefor neccesitating several weeks of indwelling time for internal stent. If we choose DJ stents with strings, the major risk of leaving the string of the stent are accidental dislodgement, and early removal of the stents by the patients. Gonen et al. showed that external ureteral catheters were more comfortable with the adult patients in a short time while being as reliable and safe as an indwelling stent (11). Vikram et al. also reported that external open-ended ureteral catheter drainage was equally effective and better tolerated than routine stenting following the uncomplicated ureteroscopic removal of stones (12). In this study, we demonstrate that the placement of external ureteral catheters instead of double-J stents, is a viable option to relieve bilateral ureteral obstruction and provide short-term urine drainage.

Most of the stones were spontaneously discharged through the bilateral catheters because of the distinctive loose and sand-like texture of ceftriaxone-associated calculi. No additional cystoscopy procedures were required after the ureteral catheters were removed.

Our study found this technique to be highly effective; moreover, there was no need for additional follow-up related to stent-related symptoms or stent removal (no lost or retained stents). This is particularly beneficial to a developing country like China, where the removal of an indwelling stent constitutes an additional procedure that is both a physical and also a financial burden on patients.

## Conclusion

In conclusion, our results showed that the application of external ureteral catheters in children with AKI caused by ceftriaxone-induced urolithiasis was effective and reliable. We recommend this procedure as a viable replacement of indwelling stents in patients with bilateral ureteral stones. However, further studies are necessary to better define the role of this method, focusing on the evaluation of symptoms and complications.

## Data Availability Statement

All datasets generated for this study are included in the article/Supplementary Material.

## Ethics Statement

The studies involving human participants were reviewed and approved by Shanghai xinhua hospital ethics committee. Written informed consent to participate in this study was provided by the participants' legal guardian/next of kin.

## Author Contributions

LH: conceived and designed the experiments. HG, MX, GX, XF, and LH: performed the experiments. MX and HL: analyzed the data. LH and GX: video making. MX: manuscript writing.

## Conflict of Interest

The authors declare that the research was conducted in the absence of any commercial or financial relationships that could be construed as a potential conflict of interest.

## Supplementary Material

Video S1 External ureteral catheterization procedure. The ureteral opening was identified using the cystoscope, and the external stent was inserted. After entering the ureter, there was a sense of resistance; after the thrust was appropriately increased, the stent continued to enter. At this time, the ureteral opening contained urine and turbid stones.

Video S2 Urine continued to flow from the end of the stent, confirming that the obstruction was relieved.
