# Corrigendum: The Application of External Ureteral Catheters in Children With Acute Kidney Injury Caused by Ceftriaxone-Induced Urolithiasis

**DOI:** 10.3389/fped.2020.00513

**Published:** 2020-09-02

**Authors:** Houwei Lin, Hongquan Geng, Guofeng Xu, Xiaoliang Fang, Lei He, Maosheng Xu

**Affiliations:** ^1^Department of Pediatric Urology, Xinhua Hospital, Shanghai Jiao Tong University of Medicine, Shanghai, China; ^2^Children's Urolithiasis Treatment Center of Chinese Health Committee, Shanghai, China

**Keywords:** external ureteral catheter, acute kidney injury, ceftriaxione, urolithiasis, children

The order of the authors was incorrectly listed as **Maosheng Xu, Hongquan Geng, Guofeng Xu, Xiaoliang Fang, Lei He and Houwei Lin**. The correct order is **Houwei Lin, Hongquan Geng, Guofeng Xu, Xiaoliang Fang, Lei He and Maosheng Xu**. The correct corresponding author is **Maosheng Xu xumaosheng@xinhuamed.com.cn**.

The authors apologize for this error and state that this does not change the scientific conclusions of the article in any way. The original article has been updated.

